# Process evaluation of the implementation of the ABC method, an intervention for nurses dealing with challenging behaviour of patients with brain injury

**DOI:** 10.1186/s12912-024-01987-w

**Published:** 2024-05-28

**Authors:** Climmy Pouwels, Peggy Spauwen, Hilde Verbeek, Ieke Winkens, Rudolf Ponds

**Affiliations:** 1 Multidisciplinary Specialist Centre for Brain Injury and Neuropsychiatry, GGZ Oost Brabant, Boekel, The Netherlands; 2 De Zorggroep Noord- en Midden-Limburg, P.O. Box 694, Venlo, 5900 AR The Netherlands; 3 Department of Psychiatry and Neuropsychology, School for Mental Health and Neuroscience (MHeNS), Maastricht University, Maastricht, The Netherlands; 4 Clinical Centre of Excellence for Personality Disorders in Older Adults, Mondriaan Mental Health Center, Heerlen-Maastricht, The Netherlands; 5 Department of Health Services Research, CAPHRI, Maastricht University, Maastricht, The Netherlands; 6 Limburg Brain Injury Centre, Maastricht University, Maastricht, The Netherlands; 7 Department of Neuropsychology and Psychopharmacology, Faculty of Psychology and Neuroscience, Maastricht University, Maastricht, The Netherlands; 8 Department of Medical Psychology, Amsterdam University Medical Centre, Amsterdam, The Netherlands

**Keywords:** Intervention, Implementation, Process evaluation, Healthcare innovation, Challenging behaviour, Nursing staff

## Abstract

**Background:**

Introducing new working methods is common in healthcare organisations. However, implementation of a new method is often suboptimal. This reduces the effectiveness of the innovation and has several other negative effects, for example on staff turnover. The aim of the current study was to implement the ABC method in residential departments for brain injured patients and to assess the quality of the implementation process. The ABC method is a simplified form of behavioural modification based on the concept that behaviour operates on the environment and is maintained by its consequences.

**Methods:**

Four residential departments for brain injured patients introduced the ABC method sequentially as healthcare innovation using a stepped-wedge design. A systematic process evaluation of the implementation was carried out using the framework of Saunders et al. Descriptive statistics were used to analyse the quantitative data; open questions were clustered.

**Results:**

The training of the ABC method was well executed and the nursing staff was enthusiastic and sufficiently involved. Important aspects for successful implementation had been addressed (like a detailed implementation plan and implementation meetings). However, facilitators and barriers that were noted were not addressed in a timely manner. This negatively influenced the extent to which the ABC method could be properly learned, implemented, and applied in the short and long term.

**Conclusions:**

The most challenging part of the introduction of this new trained and introduced method in health care was clearly the implementation. To have a successful implementation serious attention is needed to tailor-made evidence-based implementation strategies based on facilitators and barriers that are identified during the implementation process. Bottlenecks in working with the ABC method have to be addressed as soon as possible. This likely requires ‘champions’ who are trained for the job, next to an organisation’s management that facilitates the multidisciplinary teams and provides clarity about policy and agreements regarding the training and implementation of the new method. The current process evaluation and the recommendations may serve as an example for the implementation of new methods in other healthcare organisations.

**Supplementary Information:**

The online version contains supplementary material available at 10.1186/s12912-024-01987-w.

## Background

Aggressive and challenging behaviour after brain injury is common [1, 2]. It negatively influences a patient’s quality of life and puts a high burden not only on the patient and the patient’s family, but also on healthcare professionals [3, 4]. When confronted with aggressive patients, nurses experience more feelings of anger, fear, and other symptoms of post-traumatic stress disorder [5, 6]. Moreover, feelings of guilt and shame and depression are reported [6]. This all has consequences on work functioning such as job satisfaction and sick leave [5] and urges for tools that can help nursing staff to reduce challenging behaviour like aggression.

The ABC (Antecedent – Behaviour –Consequence) method developed by Cohn and colleagues [7] is such a tool. In essence, it is a simplified form of behavioural modification, based on the concept that behaviour operates on the environment and is maintained by its consequences [8]. Training nursing staff in applying behavioural interventions in everyday practice may be a potentially powerful tool for reducing challenging behaviour in a department [7].

The ABC method offers nurses tools and skills to become more aware of the factors that can cause challenging behaviour of patients, including the nurses’ own behaviour and communication style [7, 9]. A key component of the ABC method is a detailed and structured observation of the challenging behaviour every time the behaviour occurs by several persons of the nursing staff. Based on these observations, a functional assessment of the challenging behaviour is made by the nursing staff, mostly in cooperation with a psychologist. This assessment includes a fine graded description of the challenging behaviour and its antecedents and consequences. Based on this analysis, a patient- and situation-tailored intervention is made to reduce challenging behaviour. A more extensive description of the ABC method can be found in Winkens et al. [10].

Introducing new working methods is common in health care organizations. However, implementation of a new method is often suboptimal. This has several negative effects, for example on staff turnover, patient care and budget targets [11]. It also reduces the effectiveness of the innovation [12], as shown in our earlier study of the ABC method [13]. Good implementation serves as necessary preconditions to achieve the desired changes in healthcare by working with a new method [12]. Although there are different definitions of successful implementation, they all include a number of core aspects: it is about renewal or improvement, it is process-based and systematic, and the goal is to achieve a lasting change in the daily work- routines [14]. Important aspects for a successful implementation are a detailed and concrete implementation plan, awareness of the phases of the implementation (preparing, implementation and sustaining phase) and taking into account the factors that can positively or negatively influence the implementation. Following, implementation strategies can be determined [11, 15–18]. A number of conditions facilitate successful implementation: a motivated team with sufficient expertise, involvement of the team, leaders and key figures, good time planning and sufficient resources and support [17, 18]. The successful introduction of a new working method requires an ongoing process of reflection and evaluation with engagement and dialogue with the whole staff (including administration and management) [15].

In short, successful implementation is a challenging but very important aspect of any new intervention in healthcare to be effective. In the current study, a detailed and systematic process evaluation of the implementation of the ABC method in residential departments for patients with brain injury was carried out. The aim was to implement the ABC method in residential departments for patients with brain injury and to assess the quality of the implementation process.

## Method

### Setting

The study was carried out at four residential departments for patients with brain injury in the south of the Netherlands. One department of organization A (SGL = Stichting Gehandicaptenzorg Limburg) and three separate departments of organization B (De Zorggroep), departments B1, B2, B3.

### Design

The departments introduced the method as healthcare innovation using a stepped-wedge design (see Table 1). A stepped-wedge design is especially useful when effectiveness has not yet been investigated but it is predicted that the intervention will do more good than harm [19]. In a stepped-wedge design a method is sequentially introduced in the participating departments [19, 20]. Phases in which departments did not receive the training yet function as control phases (see Table 1). By the end of the study, all departments received the training.

**Table 1:** Stepped-wedge design

Department A	T0	**Training**	**T1**		**T2**		**T3**		**T4**	**T5**	
Department B1	T0		T1	**Training**	**T2**		**T3**		**T4**	**T5**	
Department B2	T0		T1		T2	**Training**	**T3**		**T4**	**T5**	
Department B3	T0		T1		T2		T3	**Training**	**T4**	**T5**	

T0 = baseline, T1 = immediately after training of department A, T2 = 12 weeks after T1, T3 = 24 weeks after T1, T4 = 36 weeks after T1, T5 = 48 weeks after T1

Cells without bold text represent control/baseline periods; Cells with bold text represent intervention periods and post-intervention periods

### Sample

All nurses with a permanent employment were invited by the research team to participate in the study. Nurses with temporary employments or who were not able to attend most of the training (e.g. due to nightshifts) were excluded from the study. Trainees were also excluded. The psychologists working at the departments and the management team of the department also were invited to participate in the study.

### Implementation process

An implementation coach with 20 years of experience led the implementation process. The implementation coach was an employee of the ABC’99 foundation, which develops and manages the ABC methodology in the Netherlands The aim of this foundation is to facilitate all healthcare workers in dealing with challenging behaviour and supporting organizations in implementation and integration of the methodology [21].

First, a format for an implementation plan was made. The format was based on the standard format of the ABC ‘99 foundation and adapted for use in the current study by the coach and the researcher (CP), taking into account important implementation factors and implementation strategies [15–18]. The goal of making an implementation plan is creating commitment and making agreements about the implementation process and the implementation strategies before starting the implementation itself. An implementation plan should be based on barriers and facilitators to change [16] that can be divided in three overarching domains with interrelationships: system (e.d. environmental context, culture), staff (e.d. commitment, skills) and intervention (e.d. supportive components such as training and feedback) [15]. The coach asked the participating departments to put together a core implementation team consisting of at least one member of the nursing staff, a psychologist and a member of the management team. Department B1, B2 and B3 had one core team for their three departments, meaning that their core team consisted of at least one member of the nursing staff from every department, one psychologist, and one member of the management team. The management team and the psychologist were informed about the ABC method and the purpose of working with it before the start of the study. They in turn informed the nursing staff participating in the core team.

The implementation coach planned a first implementation meeting (starting meeting) for each core team to prepare the implementation. During the starting meeting, each core team made an implementation plan with the coach. Nine topics were discussed in the core team: (1) objectives of the implementation of the ABC method, (2) the target group (nursing staff) and department (patients), (3) colleagues who will not work directly with the ABC method, (4) organization level (e.d. inserting the method into work and organizational processes), (5) ABC method and multidisciplinary collaboration, (6) ABC method and IT systems (e.d. digital medical records), (7) working with the ABC method in a team (e.d. motivation, feedback), (8) evaluation of the implementation process, (9) working with the ABC method in the long term (the sustaining phase according to Grol and Wensing [17, 18]). Agreements and implementation strategies were recorded in the implementation plan. The implementation plan also included an action plan containing what still needed to be arranged, who would do this and a deadline. The core team was responsible for the evaluation of the implementation and action plan.

A licensed trainer of the ABC’99 foundation provided the ABC training. The ABC training started after the first implementation meeting and was given in groups of maximally thirteen nurses. Therefore, department A, B1 and B3 were divided in two groups. Each nurse received the ABC training in five half-days in a period of five to nine weeks. The training provided the nurses information, tools and skills to become more aware of the antecedents of challenging behaviour and to deal with these problems. The training consisted of five modules each consisting of predetermined parts: communication with people with brain injury (5 parts), behavioural observation (8 parts), confused behaviour (11 parts), depressive behaviour (7 parts) and agitated behaviour (6 parts). At the end of the training, the nurses received an ABC card, which is a small card with a summary of the specific steps to take and questions to answer in the phases of observation and behavioural change. In-between the training days, nurses had to complete homework assignments to practice with the newly acquired skills. A psychologist was involved in each department. The psychologist of department B1, B2, B3 participated in the training, but the psychologist of department A didn’t. Other team members (e.g. doctors) did not participate in the ABC training. They were updated with information about the ABC method by the manager and in multidisciplinary consultations.

Each core team had three additional implementation meetings with the coach. In each meeting, the members of the core teams evaluated the implementation of the method with a questionnaire and adjusted the implementation plan (including action plan) and interventions if needed. For department A and B1, these meetings took place after the ABC training was completed. For department B2 one meeting and for departmert B3 two meetings with the coach took place before the ABC training was completed due to the stepped wedge design. Therefore, an extra meeting with their members of the core team but without the coach was planned after the ABC training was completed.

### Data collection

#### Patients

To be able to describe the setting and the patients living in the participating departments, the following data were collected at baseline from patient’s files: age (years), gender, type of brain injury, time since injury and psychiatric history of the patients. The Frontal Assessment Battery (FAB) [22] and the Montreal Cognitive Assessment (MoCA) [23] were administered once by a psychologist to indicate the (cognitive) functioning of the patient population. A score below 12 (range 0–18) indicated cognitive impairments on the FAB [22] and a score below 26 (range 0–30) indicated cognitive impairments on the MoCA [23]. The Care Dependency Scale (CDS) [24] was administered once by the nurses to indicate the need and care dependency of the patients. The higher the score, the less dependent in care (range 15–75, below 69 indicates to be dependent in care). Last, the Neuropsychiatric Inventory-Questionnaire (NPI-Q) was administered at baseline by the nurses to indicate the neuropsychiatric symptoms of the patients in the last month. Only the total score was used. The higher the score, the more neuropsychiatric symptoms (range from 0 to 60). The NPI-Q is an adaptation of the Neuropsychiatric Inventory (NPI) [25] and has been validated [26]. Of all questionnaires, the Dutch version was used.

#### Nurses

For each participating nurse, gender, age (years), highest educational level, working experience (in years) and working experience at the department (in years) were obtained once at baseline via a digital survey. Nurses received a link for this digital survey via their email address. Education was assessed according to the Dutch school system and subsequently compared with The European Qualifications Framework [27].

#### Process evaluation

The quality of the implementation was assessed with a process evaluation based on the framework of Saunders et al. [28]. The training was evaluated separately using the same framework. The framework consists of three main elements (see Table 2). First the extent to which the ABC method was trained and implemented as planned (fidelity, dose delivered), second the exposure and satisfaction with the training and implementation of the ABC method (dose received, reach) and third the influence of the context (barriers) on the training and the implementation of the ABC method. All data (each item of the questionnaires) were assessed by the authors and subdivided into the three main elements of the model of Saunders et al. [28]. See the tables to know which items belong to which element.

**Table 2:** Measurement method based on the framework of Saunders et al. [28]

Concept	Operationalization							
		A	L	ETN	IP	IQN	IQC	ERM
According to plan (fidelity and dose delivered)	The extent to which all modules and parts of the training were deliverd.		X					
	The extent to which the ABC method was implemented as planned				X		X	X
Exposure and satisfaction (reach and dose received)	Proportion of the nurses that attended the training and their active involvement	X	X					
	The extent to which the ABC method is used					X	X	X
	Disciplines attended the implementation meetings				X			X
	Satisfaction with the training, the ABC method and the implementation proces			X		X	X	X
Barriers (context)	Barriers of the training, working with the ABC method and the implementation.			X	X	X	X	X
	The extent to which the barriers/problems were solved.				X			X

A = Attendance list by the trainer, L = Logbook of the training by the trainer, ETN = Evaluation of the training by the nursing staff, IP = Implementation plan by the core team and the coach, IQN = Implementation questionnaire nursing staff, IQC = Implementation questionnaire core team, ERM = Evaluation report of the meetings of the core team

The following data were collected (see Table 2 for an overview):

- After each ABC training day, the trainer completed the attendance list (AT) and completed a logbook (L) of whether all parts of the module were covered and whether everyone had done their homework (and if not, why not) (see Table 3).
- At the end of the ABC training, all nurses completed a non-validated questionnaire (ETN) developed and used as a standard by the ABC’99 foundation. The questionnaire aimed at evaluating the training by the nurses and consists of rating five aspects of the training from 1 to 10 and some open questions. The research team added some extra questions to asses some other aspects like the complexity of the training and the time to do their homework answered on a 5-point Likert-scale (see additional file 1 and Table 4).
- To evaluate the implementation process we checked whether each department had made an implementation plan (IP), whether all nine topics of this plan were discussed (e.g. objectives of the implementation of the ABC method, ABC method and multidisciplinary collaboration, ABC method and IT), which issues still need to be addressed and whether all additional implementation meetings took place.
- Two non-validated questionnaires were used to evaluate the quality of the implementation, one for the nurses and one for the core team. The questionnaires were based on of the format of the implementation plan taking into account important implementation factors and implementation strategies [15–18].
  The questionnaire for the nurses (IQN) consisted of seven statements (e.g. everyone works in de the same way according to the ABC method) answered on a 5-point Likert-scale and one question (how enthusiastic are you about working with the ABC method?) rating from 1 to 10 (see Additional file 2 and Table 5). Nurses completed the implementation questionnaire immediately after the training and then again every 12 weeks. Due to the stepped wedge design, it differs per department how often the questionnaire was administered. Nurses from department A completed the questionnaire 4 times due to missing data at T5 (T1, T2, T3, T4), Department B1 completed the questionnaire also 4 times (T2, T3, T4, T5), department B2 completed the questionnaire 3 times (T3, T4, T5), and department B3 completed the questionnaire 2 times (T4, T5).
  The members of the core teams filled out another questionnaire (IQC) every additional implementation meeting. This questionnaire consisted of thirteen statements answered on a 5-point Likert-scale, five yes/no questions (e.g. the ABC method is a regular topic in multidisciplinary discussions) and one question (how enthusiastic is the team about working with the ABC method?) rating from 1 to 10 (see Additional file 3 and Table 6).
- The implementation coach made a descriptive evaluation report (ERM) of each additional implementation meeting.

**Table 3:** Evaluation of the ABC training by the trainer

			Dept. A	Dept. B1	Dept. B2	Dept. B3
Training given as planned	Doses delivered	Modules discussed (%)	100%	100%	100%	100%
		Parts discussed*	Group1 / Group2	Group1 / Group 2		Group1 / Group2
		• Module 1 (5 parts)	5 / 5	5 / 5	5	5 / 5
		• Module 2 (8 parts)	8 / 8	8 / 8	8	7 / 8
		• Module 3 (11 parts)	11 / 10	10 / 10	8	11 / 11
		• Module 4 (7 parts)	7 / 6	7 / 7	6	7 / 7
		• Module 5 (6 parts)	6 / 6	6 / 6	5	6 / 6
Exposure and satisfaction with the training	Doses received	Homework done (%)				
		• Meeting 2	71.4	90.5	91.7	100
		• Meeting 3	^	100	77.3	100
		• Meeting 4	~	90.9	65	69
		• Meeting 5	~	81.8	33.3	~
	Reach	Percentage of nurses that attended at least 80% of the training	85.60%	87.20%	83.10%	73.10%

Dept. = department

* The ABC training was given in groups of maximally thirteen nurses. Therefore, department A, B1 and B3 were divided in two groups.

^ Trainer described % homework done as ‘almost nobody’

∼ missing

**Table 4:** Evaluation of the ABC training by the nurses (ETN)

			Dept.A *N* = 14 Median (IQR)	Dept.B1 *N* = 11 Median (IQR)	Dept.B2 *N* = 6 Median (IQR)	Dept.B3 *N* = 22 Median (IQR)
Training given as planned	Dose delivered	I am informed well about the training (1 = totally disagree to 5 = totally agree)	3.00 (1.75–4.25)	4.00 (3.00–4.00)	4.00 (3.75-5.00)	3.00 (2.00–4.00) M = 4
Exposure and satisfaction with the training	Dose received	Training as a whole (1 = very bad to 10 = very good)	7.50 (7.00-8.25)	7.00 (6.00–7.00)	6.50 (6.00–8.00)	7.00 (6.00–7.00)
		Applicability in work (1 = very bad to 10 = very good)	7.00 (6.75–7.25)	7.00 (5.00–8.00)	6.00 (6.00-7.25)	6.00 (5.75-7.00)
		Trainer (1 = very bad to 10 = very good)	9.00 (8.00–9.00)	8.00 (7.00–8.00)	7.00 (6.75–7.25)	7.00 (7.00-8.25)
		Accommodation of the training (1 = very bad to 10 = very good)	7.00 (6.75-8.00)	6.00 (5.00–7.00)	5.50 (3.75–6.25)	5.00 (3.75-7.00) M = 4
		Working methods (1 = very bad to 10 = very good)	7.00 (7.00-8.25)	7.00 (5.00–8.00)	6.50 (6.00-7.25)	6.50 (6.00–7.00)
		The training was educational (1 = totally disagree to 5 = totally agree)	4.00 (4.00–5.00)	4.00 (3.00–4.00)	3.50 (3.00-4.25)	3.00 (3.00–4.00) M = 4
		I understood the training (1 = totally disagree to 5 = totally agree)	4.00 (4.00–5.00)	4.00 (4.00–5.00)	5.00 (4.75-5.00)	4.00 (4.00–5.00) M = 4
		The training was too complicated (1 = totally disagree to 5 = totally agree)	1.00 (1.00–2.00)	1.00 (1.00–2.00)	1.00 (1.00–2.00)	1.00 (1.00–2.00) M = 4
Barriers on the training	Context	I had enough time to do homework (1 = totally disagree to 5 = totally agree)	2.00 (1.75–3.25)	4.00 (3.00–5.00)	4.50 (3.00–5.00)	4.50 (4.00–5.00) M = 4
		I could appeal to colleagues when I had problems with my homework (1 = totally disagree to 5 = totally agree)	4.00 (3.00–4.00)	5.00 (4.00–5.00)	4.50 (3.75-5.00)	4.50 (4.00–5.00) M = 4
		I could appeal to the trainer when I had problems with my homework (1 = totally disagree to 5 = totally agree)	4.00 (3.00–4.00)	4.00 (3.00–4.00)	3.00 (2.75–4.25)	3.00 (2.00–5.00) M = 5
		I want a booster session in the future (1 = totally disagree to 5 = totally agree)	4.00 (3.00–5.00)	2.00 (1.00–4.00)	3.00 (2.75-3.00)	3.00 (2.75–3.13) M = 4

Dept. = department

M = missing

**Table 5:** Evaluation of the implementation by the nurses at the last measurement point (ICN)*

			Dept.A *N* = 10 Median (IQR)	Dept.B1 *N* = 11 Median (IQR	Dept.B2 *N* = 11 Median (IQR)	Dept.B3 *N* = 13 Median (IQR)
Implementation as planned	Dose delivered	It is clear to me why we started working with the ABC method (1 = totally disagree to 5 = totally agree)	4.00 (4.00–4.00)	4.00 (3.00–5.00)	3.00 (3.00–4.00)	3.00 (2.00–4.00)
Exposure and satisfaction with the implementation	Doses received	I have sufficient knowledge and skills to work with the methodology after the training (1 = totally disagree to 5 = totally agree)	4.00 (3.00–4.00)	4.00 (4.00–4.00)	3.00 (3.00–4.00)	4.00 (2.50-4.00)
		How enthusiastic are you about working with the ABC method? (1 = not at all to 10 = totally)	6.50 (3.73–7.25)	7.00 (7.00–8.00)	6.00 (5.00–7.00)	5.00 (3.00–5.00)
		Working with the ABC method is routine (1 = totally disagree to 5 = totally agree)	1.50 (1.00–3.00)	4.00 (3.00–4.00)	2.00 (2.00–3.00)	2.00 (2.00-2.50)
		Working with the ABC method has reduced challenging behaviour on the department (1 = totally disagree to 5 = totally agree)	3.00 (1.75-3.00)	3.00 (2.00–3.00)	2.00 (2.00–3.00)	2.00 (1.00–2.00)
Barriers to the implementation	Context	Everyone works in the same way according to the ABC method (1 = totally disagree to 5 = totally agree)	3.00 (1.75-3.00)	4.00 (4.00–4.00)	3.00 (3.00–4.00)	2.00 (2.00–3.00)
		There is sufficient time to work with or discuss the ABC method (1 = totally disagree to 5 = totally agree)	2.50 (1.00–3.00)	4.00 (3.00–4.00)	3.00 (3.00–4.00)	2.00 (2.00–3.00)
		There is sufficient support from a therapist or psychologist (1 = totally disagree to 5 = totally agree)	1.00 (1.00–3.00)	4.00 (4.00–4.00)	4.00 (3.00–4.00)	3.00 (3.00–4.00)

Dept. = department

*Dept. A = T4, Dept. B1, B2, B3 = T5

**Table 6:** Evaluation of the implementation by the questionnaires of the core team (IQC)

			Dept.A *N* = 5 Meeting 3	Dept.B1, B2, B3 *N* = 8 Meeting 2^*^
Implementation as planned	Dose delivered	It is clear to the whole team why we started working with the ABC method. (1 = totally disagree to 5 = totally agree) (Median (IQR))	4.00 (3.50-4.00)	4.00 (3.00–4.00) M = 1
		The theory about the ABC method is regularly discussed and repeated in the team. (1 = totally disagree to 5 = totally agree) (Median (IQR))	3.25 (2.25–3.88) M = 1	3.00 (1.50–3.75) M = 4
		Does anyone know where in the (electronic) file they can report about the ABC method? (yes %)	50% M = 1	60% M = 3
		Team members actively train new colleagues or students in the ABC method. (yes %).	33.3% M = 2	0% M = 3
		A trainer of the ABC’99 foundation trains new colleagues or student. (yes %)	0%	0% M = 3
Exposure and satisfaction with the implementation	Doses received	After the training, the team will have sufficient knowledge to work with the methodology. (1 = totally disagree to 5 = totally agree) (Median (IQR))	4.00 (3.00–4.00)	3.00 (3.00–4.00) M = 2
		How enthusiastic are you about working with the ABC method? (1 = not at all to 10 = totally) (Median (IQR))	5.50 (3.50–6.75) M = 1	6.50 (5.50-7.00) M = 3
		The (electronic) files shows that the ABC method is used. (1 = totally disagree to 5 = totally agree) (Median (IQR))	3.50 (1.50-4.00) M = 1	4.00 (2.50-5.00) M = 3
		There is discussion about disagreements and after that, a hypothesis about the behavioural problem and an action plan is drawn up together. (1 = totally disagree to 5 = totally agree) (Median (IQR))	3.00 (2.00-3.50)	3.00 (3.00-4.25) M = 2
		Team members give each other sufficient feedback about working with the ABC method. (1 = totally disagree to 5 = totally agree) (Median (IQR))	2.50 (2.00-3.75) M = 1	2.50 (2.00-3.75) M = 4
		Working with the ABC method has reduced challenging behaviour on the department. (1 = totally disagree to 5 = totally agree) (Median (IQR))	2.00 (2.00-3.50) M = 1	3.00 (1.50–3.75) M = 4
		Working with the ABC method is routine. (1 = totally disagree to 5 = totally agree) (Median (IQR))	2.00 (1.25–2.75) M = 1	2.50 (1.25–3.75) M = 4
Barriers to the implementation	Context	Everyone works in the same way according to the ABC method. (1 = totally disagree to 5 = totally agree) (Median (IQR))	3.00 (2.00-3.50)	3.00 (2.00–3.00) M = 3
		There is sufficient time to work with or discuss the ABC method. (1 = totally disagree to 5 = totally agree) (Median (IQR))	3.00 (1.50-3.00)	4.00 (3.00–4.00) M = 1
		The ABC method is a regular topic in daily discussions about a client. (yes %)	100% M = 1	33.3% M = 2
		The ABC method is a regular topic in multidisciplinary discussions. (yes %)	66.7% M = 2	0% M = 2
		There is sufficient support from a therapist or psychologist. (1 = totally disagree to 5 = totally agree) (Median (IQR))	3.00 (2.00-3.50)	4.00 (4.00–5.00) M = 1
		Successes related to working with the ABC method are celebrated (compliments, attention to positive changes). (1 = totally disagree to 5 = totally agree) (Median (IQR))	2.00 (1.25–2.75) M = 1	3.50 (1.50-4.00) M = 4
		Bottlenecks in working with the ABC method are identified and addressed in a timely manner. (1 = totally disagree to 5 = totally agree) (Median (IQR))	2.50 (2.00–3.00) M = 1	3.00 (1.50-3.00) M = 4

Dept. = department

M = missing

*= Questionnaires of meeting 3 were missing, the member of B3 did not have the training at the moment of meeting 2

### Analyses

Descriptive statistics were used to describe the characteristics of the nursing staff, the patient population, the evaluation of the training and the evaluation of the implementation. There were some open questions. Open questions that provided additional information were clustered. All statistical analyses were conducted with the use of IBM SPSS version 26.

## Results

### Demographic characteristics of the patients

A total of 45 patients were living at the departments, 19 at department A, six at department B1, 11 at department B2 and nine at department B3. In summary, the patients were most often males (with the exception of department B3), who had challenging behaviour, were severely cognitively impaired and dependent on care. The types of brain injury (contusion cerebri and brain injury due to vascular incidents were most common) varied greatly between the departments. Details per department can be seen in Table 7.

**Table 7:** Demographic characteristics of the patients

	Dept. A (*N* = 19*)	Dept. B1 (*N* = 6**)	Dept. B2 (*N* = 11***)	Dept. B3 (*N* = 9)
Age in years (m(sd))	58.8 (10.4)	55.7 (12.7)	55.9 (9.3)	52.3 (9.7)
Gender (M/F %)	82/18	100/0	91/9	56/44
Psychiatric history (Yes in %)	53	100	36	22
MoCA (m(sd))	16.6 (7.3)	16.6 (5.1)	17.3 (4.8)	16.0 (5.2)
FAB (m(sd))	13.4 (4.9)	12.0 (2.8)	13.1 (1.2)	10.7 (4.1)
CDS (m(sd))	47.0 (15.8)	50.5 (20.6)	45.1 (17.8)	52.2 (14.6)
NPI severity (m(sd))	9.1 (6.7)	7.3 (5.6)	9.5 (5.0)	6.9 (4.3)
NPI distress caregiver (m(sd))	8.4 (8.7)	10.8 (8.9)	10.0 (7.3)	4.9 (6.8)

Dept. = department, MoCA = Montreal Cognitive Assessment, FAB = Frontal Assessment Battery, CDS = Care Dependency Scale, NPI = Neuropsychiatric Inventory

*Missing A: Age and Gender *N* = 2, Type of Injury *N* = 1, FAB *N* = 11, MoCA *N* = 9, Type of injury *N* = 1

** Missing B1: Gender *N* = 1, FAB and MoCA *N* = 1

*** Missing B2: FAB and MoCA *N* = 1

### Demographic characteristics of the nursing staff

A total of 61, mostly female, nurses with mostly intermediate vocational education (EQF 2 to 4 according to The European Qualifications Framewor [27]) participated in the study at baseline, 22 at department A, 14 at department B1, 11 at department B2 and 14 at department B3. Nurses in all departments had on average more than ten years of working experience. However, the nurses of department A had the fewest years of working experience. The nurses of department A and B3 were younger than the nurses of department B1 and B2 (see Table 8).

**Table 8:** Demographic characteristics of the nursing staff

	Dept. A (*N* = 22)	Dept. B1 (*N* = 14)	Dept. B2 (N = 11)	Dept. B3 (N = 14)
Age in years (m(sd))	37.6 (11.8)	45.5 (11.0)	47 (7.4)	38.0 (11.8)
Gender (M/F %)	0/100	14.3/85.7	18.2/81.8	0/100
Educational level*				
1	0	0	0	0
2	0	0	1	0
3	2	3	0	1
4	5	2	2	3
5	10	8	5	9
6	0	0	2	0
7	4	1	1	1
8	1	0	0	0
Years of working experience (m(sd))	11.8 (9.9)	17.8 (13.0)	20.2 (10.6)	15.8 (13.3)
Number of years working at the current department (m(sd)	4.8 (3.9)	7.3 (7.5)	14.8 (11.5)	10.2 (10.8)

Dept. = department

* 1. = no education, 2 = primary education, 3 = low vocation education, 4 = lower general secundary education, 5 = intermediate vocational education, 6 = higher general secundary education or pre university education, 7 = Higher vocational education, 8 = university degree

### Procesevaluation

The process evaluation is based on the framework of Saunders et al. [28] divided into the three main elements (see Table 2). First, the ABC training was evaluated. Subsequently, the implementation of the ABC method was evaluated.

### ABC training

#### 1. Was the training given as planned

The training logbook (L) showed that all five modules of the ABC training were discussed in all groups. In all departments, more than 90% of the parts of each module were fully discussed (see Table 3). The questionnaire used to evaluate the ABC training (ETN) showed that most nurses indicated they were sufficiently informed about the goal of the training (see Table 4).

#### 2. Exposure and satisfaction with the training

According to the training logbook (L), most nurses of department A, B1 and B2 attended at least 80% of the training. In department B3 this percentage was slightly lower. The percentage of nurses that had completed their homework varied per department and over time. The percentage of nurses who did their homework was lowest at department A. The main reason for not doing their homework was too little time. After meeting 3, the trainer no longer noted the exact number of people who did or did not do their homework (see Table 3).

Results from the nurses’ questionnaire on evaluation of the training (ETN) showed that most nurses were satisfied with the trainer, the training, the complexity and the degree of applicability in the work. In general, department A was most satisfied (see Table 4).

#### 3. Barriers

The logbook (L) showed that the main reasons for not attending a meeting were being ill, having a holiday and a work schedule (e.g. night shift) that did not match the date and time of the meeting. According to the trainer, too little time for homework (partly because of holidays) was the main reason why homework was not done. This was only confirmed by the questionnaire of the nursing staff (ETN) at department A. All departments indicated that they could appeal to their colleagues (slighly lower for department A) and trainer (lowest for department B2 and B3) in case of problems. The majority of nurses indicated they would like a booster session of the ABC method, department A more than departments B1, B2 and B3 (see Table 4).

### Implementation of the ABC method

#### 1. Was the ABC method implemented as planned

All departments made an implementation plan (IP) in which the 9 predefined topics were described. However, topic 9 (working with the ABC method in the long term) was only briefly discussed. In addition, facilitators and barriers of the teams were discussed in the first meeting and included in the implementation plan (IP), but no specific interventions were initiated for barriers such as bad experiences with the effectiveness of training in the past resulting in little motivation, difficulty giving feedback to each other, no implementation experience, much impact on staff scheduling (see Additional file 4 for a complete overview of the barriers discussed during the meetings). For all departments, additional implementation meetings were planned and took place together with the implementation coach except the extra meetings of department B2 and B3.

According to the implementation questionnaires of the nursing staff (IQN) and the core team (IQC) everyone was sufficiently informed about the reason to start working with the ABC method. The nursing staff of department B3 indicated to the implementation coach that they were insufficiently informed about the implementation itself. The theory about the ABC method was sufficiently discussed at all departments according to the core team and repeated in the teams. However, only 50% of the core team of department A and 60% of the core team of department B1, B2 and B3 agreed with the statement ‘everyone knows where in the (electronic) file they can report about the ABC method’. According to the entire core team, a licensed trainer did not train new colleagues and only one member of the core team of department A agreed with the statement that new colleagues were trained in the ABC method by team members (see Tables 5 and 6).

#### 2. Exposure and satisfaction with the implementation of the ABC method

At the start meeting, when the implementation plan (IP) was made, of all departments all disciplines were present. This was not the case at the implementation meetings, but a broad representation was generally present.

According to the implementation questionnaires (IQN and IQC) the nursing staff and the core team indicated they had sufficient knowledge and skills after the training to start working with the ABC method. With the exception of department B1, the nursing staff was not so enthusiastic about working with the method and according to the nursing staff there is no clear decrease in challenging behaviour since working with the method. With the exception of the nursing staff of department B1, everyone indicated that working with the ABC method is not yet routine. However, the ABC method is sufficiently used according to the core team (see Tables 5 and 6).

#### 3. Barriers

According to the implementation questionnaires (IQN and IQC) department A and B3 indicated they had not enough time to work with or discuss the ABC method and not everyone worked in the same way according to the ABC method. Furthermore, department A indicated they had not sufficient support from a psychologist (see Table 5.) confirmed by the implementation coach (see Additional file 4). Department B1 and B2 were in generally more positive (see Table 5). According to the core team, the ABC method was a regular topic in daily multidisciplinary discussions at department A. On the other hand, this was not or hardly the case at department B1, B2 and B3. According to the implementation plan en the implementation meetings, points for improvement arising from the implementationplan were not carried out before or during the training (see Additional file 4). In addition, bottlenecks in working with the ABC method were identified and addressed to a limited extent according to the core team and the coach (see Table 6).

## Discussion

Successful implementation is a challenging but very important aspect of any new intervention in healthcare [12]. The objective of the current study was to describe and assess the quality of the implementation process of the ABC method at four residential departments for patients with brain injury, severe cognitive impairments and challenging behaviour. The ABC method is a simplified form of behavioural modification for the nursing staff to deal with behavioural problems [7].

The departments introduced the ABC method sequentially as healthcare innovation using a stepped-wedge design [19, 20]. The training and the implementation were assessed seperately with a process evaluation based on the framework of Saunders et al. [28]. The framework consists of three main elements: first the extent to which the ABC method was trained and implemented as planned, second the exposure and satisfaction with the training and implementation of the ABC method and third the influence of the context (barriers) on the training and the implementation of the ABC method.

The results show that, overall, the training of the ABC method was carried out as planned. Overall nurses were satisfied with the trainer, the training and the degree of applicability of the training in their work. Reported barriers such as illness, and a competitive work schedule were not related to discontent about the training itself.

The results further show that implementation of the ABC method was however not fully carried out as planned. A main issue was that intended actions resulting from noted facilitators in the implementation plan were not all carried out and interventions based on possible barriers were not initiated. Regarding the satisfaction with the implementation, overall, the nursing staff was not so enthusiastic about working with the method. In addition, the ABC method did not become a routine in daily practice. Regarding the barriers that influence the implementation it was found that teams did not have enough time to work with the new method and the method was not a regular topic in team meetings.

Important to note is that differences in results were seen between the four departments, even though for all departments the training was carried out as planned and intensity of patient care was equal across departments. Department A for example was most satisfied with the trainer and the training. Department B1 however was most enthusiastic about working with the ABC method in daily practice. Moreover, department B1 mentioned working with the ABC method was routine. This department was also most positive about working in the same way according to the ABC method, the support of a psychologist and especially the amount of time to work with the method and to discuss with each other. So although department A was most enthousiastic about the training of the ABC method, unaddressed barriers (e.g. not enough time to work with or discuss the ABC method, insufficient support from a psychologist) seem to have negatively influenced the implementation of the ABC method on the work floor.

A possible explanation for the differences in the results between departments could be that the nurses of department A were significantly younger and had less working experiences (albeit this difference in working experience was not significant). In the study of van der Heijden et al. [29] it was found that younger nurses experience signficantly more emotional and physical demands, perceived stress, and developmental opportunities than older nurses. As a result, the nursing staff of department A possibly had a greater need for knowledge and skills (and were likely more eager to be trained in working with new methods) but also are more likely to experience barriers in working with new methods, and hence need more time to practice and more support. Therefore, the implementation of a new trained method might be challenging, especially when the nursing staff is young and has less working experiences.

Another possible explanation for the difference between the departments B1, B2 and B3 could be that department B2 and B3 had an extra implementation meeting due to the stepped wedge design but without the coach which could had a negative effect. Furthermore, they had one core team. Despite many things in common, it is likely that motivation, culture and working agreements differ per department. Perhaps each department should have had its own core team.

Overall, our process evaluation based on the framework of Saunders et al. [28] shows that the training of the ABC method was well executed. Important aspects and interventions for successful implementation have been addressed like an implementation plan including conditions that could facilitate a successful implementation (e.g. involvement of the team, leaders and key figures, good time planning) and factors that can positively (faciliatators) or negatively (barriers) influence the implementation. Furthermore, implementation meetings were planned [11, 15–18]. However, facilitators and barriers that were noted were not addressed in a timely manner. This negatively influenced the extent to which the ABC method could be properly learned, implemented, making it a routine and working with the method in the long term [30]. Subsequently, this had a negative influence on the satisfaction of working with the method and the perceived effectiveness of the method [12].

From the literature, we know that the use of implementation strategies carefully selected for the identified barriers and bottlenecks is neccessary to implement a new working process or method [31, 32] and to keep using the method [33]. The selection of the implementation strategies schould be tailor-made and based on the facilitators, the barriers, the method or intervention and the context (e.g. the organisation) [32] as also seen in previous studies [34]. This selection should be included in an implementation plan [32]. Implementation Mapping is a practical method that could provide a systematic process for developing and selecting implementation strategies [32, 33].

Although we tried to adopt some of these important notes, our implementation was not succesfull. In hindsight, we think that so-called ‘champions’ were lacking in the trained departments. A ‘champion’ is a leader, who fosters and reinforces changes for improvement, a facilitator of success [35, 36]. They take action in response to bottlenecks, facilitators and barriers. According to the review of Woo et al. [36] ‘champions’ are identified as one of the single most effective implementation strategies. In our study, key figures were involved for each department. However, to be able to become a true ‘champion’, key figures need to be specifically trained in their role and be facilitated by the organisation’s management. This was not sufficiently done in our study. In addition to facilitate the ‘champion’ the organisations’ management also has to facilitate the nursing staff in participating in the ABC training, getting enough time to work with the method and discussing it with each. Furthermore, the management should facilitate other team members, such as a psychologist, to be able to support the nursing staff in working with a new method. The organisational management has to provide clarity about the policy and agreements to avoid confusion [35].

### Limitations

This is the first study examining the training and the implementation of the ABC method using a structured process evaluation. Some limitations of the study need to be discussed. First, As far as we know there are no validated questionnaires to evaluate implementation of new methods on the work floor. So, self-developed questionnaires were used. These questionnaires were based on formats of the ABC ’99 foundation; however no validation took place (e.g. checking comprehensibility with the target group). This requires some caution interpreting the results. Second, there are missing values in our data due to staff leaving during the course of the study or due to illness at the time of training or assessment. Third, due to missing values, we did not have data on the evaluation of the implementation by the core team (including at least one member of the nursing staff, one psychologist, and one member of the management team) at implementation meeting 3. So, data of implementation meeting 2 were used. However, department B3 had not yet been trained at that moment due to the stepped wedge design. As a result, the evaluation of the implementaion by the core team was only based on department B1 and B2.

## Conclusion

In conclusion our findings clearly demonstrate how difficult implementation of new working methods in residential departments for patients with brain injury is. The training of a new method is the ‘easy part’ and it is not difficult to create enthusiasm and involvement of the nursing staff. The most challenging part is, however, the implementation of the new trained method at the departments following the training. To have a successful implementation serious attention is needed to tailor-made evidence based implementation strategies based on the facilitators and the barriers that are noted before the start of the implementation in the implementation plan. Interventions for motivation and culture of a team should certainly not be forgotten. Furthermore, bottlenecks in working with the ABC method have to be addressed as soon as possible. As a next step, we think ‘champions’ are needed who are trained for the job, next to an organisation’s management that facilitates the multidisciplinary team (nursing staff, psychologist) and provides clarity about policy and agreements regarding the training and implementation of a new method. Last, to be able to perform a well-designed and high-quality evaluation of an implementation process, tailored questionnaires need to be developed and validated (e.g. consultation with a trainer regarding completeness and performing pre-tests to check comprehensibility with the target group).

### Electronic supplementary material

Below is the link to the electronic supplementary material.

Supplementary Material 1

## Data Availability

The data that support the findings of the current study are available from the corresponding author on reasonable request.
