# Supplementary material for: Process evaluation of the implementation of the ABC method, an intervention for nurses dealing with challenging behaviour of patients with brain injury
Source: BMC Nurs. 2024 May 28;23:354. doi: 10.1186/s12912-024-01987-w (PMC11131173; doi:10.1186/s12912-024-01987-w)
Supplement: Supplementary file 1 — Supplementary Material 1 [file 12912_2024_1987_MOESM1_ESM.docx]

Additional file 1 Evaluation of the training by the nursing staff (ETN)

Department:

Name:

Number of meetings present:

Give your rating by circling the number. 1 = very bad to 10 = very good

1. The training as a whole

1 2 3 4 5 6 7 8 9 10

2. The trainer

1 2 3 4 5 6 7 8 9 10

3. The working methods

1 2 3 4 5 6 7 8 9 10

4. Applicability in work

1 2 3 4 5 6 7 8 9 10

5. The accommodation of the training

1 2 3 4 5 6 7 8 9 10

What did you learn from the training?

Comments?

I’m informed well about the training. Yes/No (Delete what is not applicable). If no, what information did you receive and why was it insufficient?

Circle the number that represents your opinion about the statement.

1 = totally disagree to 5 = totally agree

1. The training was educational

1 2 3 4 5

2. I’m informed well about the training

1 2 3 4 5

3. I want a booster session in the future

1 2 3 4 5

4. I had enough time to do homework

1 2 3 4 5

5. I could appeal to colleagues when I had problems with my homework

1 2 3 4 5

6. I could appeal to the trainer when I had problems with my homework

1 2 3 4 5

7. I understood the training

1 2 3 4 5

Additional file 2 Questionnaire on the evaluation of the implementation by the nurses (IQN)

Name:

Date:

Circle the number that represents your opinion about the statement.

1 = totally disagree to 5 = totally agree

1. It is clear to me why we started working with the ABC method

1 2 3 4 5

2. After the training, will I have sufficient knowledge and skills to work with the methodology

1 2 3 4 5

3. Everyone works in the same way according to the ABC method

1 2 3 4 5

4. There is sufficient time to work with or discuss the ABC method

1 2 3 4 5

5. There is sufficient support from a therapist or psychologist

1 2 3 4 5

6. Working with the ABC method is routine

1 2 3 4 5

7. Working with the ABC method has reduced challenging behaviour on the department

1 2 3 4 5

Give a rating for the next question. Circle the number that applies to you.

8. How enthusiastic are you about working with the ABC method?

1 2 3 4 5 6 7 8 9 10

Comments:

Additional file 3 Questionnaire on the evaluation of the implementation by the core team (IQC)

Name:

Date:

Circle the number that represents your opinion about the statement.

1 = totally disagree to 5 = totally agree

1. It is clear to the whole team why we started working with the ABC method

1 2 3 4 5

2. After the training, the team will have sufficient knowledge to work with the methodology

1 2 3 4 5

3. Everyone works in the same way according to the ABC method

1 2 3 4 5

4. There is sufficient time to work with or discuss the ABC method

1 2 3 4 5

5. There is sufficient support from a therapist or psychologist

1 2 3 4 5

6. The theory about the ABC method is regularly discussed and repeated in the team

1 2 3 4 5

7. Team members give each other sufficient feedback about working with the ABC method

1 2 3 4 5

8. There is discussion about disagreements and after that, a hypothesis about the behavioural problem and an action plan is drawn up together

1 2 3 4 5

9. The (electronic) files shows that the ABC method is used

1 2 3 4 5

10. Successes related to working with the ABC method are celebrated (compliments, attention to positive changes)

1 2 3 4 5

11. Bottlenecks in working with the ABC method are identified and addressed in a timely manner

1 2 3 4 5

12. Working with the ABC method is routine

1 2 3 4 5

13. Working with the ABC method has reduced challenging behaviour on the department

1 2 3 4 5

Answer the following question with yes or no, circle the correct answer.

14. The ABC method is a regular topic in multidisciplinary discussions.

Yes No

15. The ABC method is a regular topic in daily discussions about a client.

Yes No

16. Does anyone know where in the (electronic) file they can report about the ABC method?

Yes No

17. Team members actively train new colleagues or students in the ABC method.

Yes No

18. A trainer of the ABC’99 foundation trains new colleagues or students.

Yes No

Give a rating for the next question. Circle the number that applies to you.

19. How enthusiastic are the nurses about working with the ABC method?

1 2 3 4 5 6 7 8 9 10

Comments:

Additional file 4 Barriers to the implementation according the meetings with the core team and the coach

|  |  |  | **Dept. A** | **Dept. B1** | **Dept. B2** | **Dept. B3** |
| --- | --- | --- | --- | --- | --- | --- |
| Barriers to the implementation | Context | Things to do according the implementation plan and when are these things done | Interventions on the predetermined limiting factors    ABC method in the ECD (meeting 3)  Informing colleagues other than the nursing staff  Informing family’s  ABC method introduced in mdo  (meeting 3)  Making agreements about working with the ABC method in the long term | Interventions on the predetermined limiting factors  ABC method in the ECD/agreements about how and where to report in the ECD (meeting 2)  Informing family’s  Implementation of the ABC method introduced in team meeting  Making agreements about working with the ABC method in the long term | | |
|  |  | What does the coach notice | Psychologist not actively involved at the start  Working agreements were not clear  Many colleagues were sick  New colleagues were started who have not been trained  Despite the above, the method is used, but the team's capacity is limited | They are predominantly positive about working with the ABC method  Team members think differently about the ABC method (enthusiastic versus no added value) | | Team was unsatisfied with the communication about the implementation |

*Dept. = department*
